# C3aR and C5aR1 act as key regulators of human and mouse β-cell function

**DOI:** 10.1007/s00018-017-2655-1

**Published:** 2017-09-18

**Authors:** Patricio Atanes, Inmaculada Ruz-Maldonado, Attilio Pingitore, Ross Hawkes, Bo Liu, Min Zhao, Guo Cai Huang, Shanta J. Persaud, Stefan Amisten

**Affiliations:** Diabetes Research Group, Division of Diabetes and Nutritional Sciences, Faculty of Life Sciences and Medicine, Hodgkin Building, King’s College London, Guy’s Campus, London, SE1 1UL UK

**Keywords:** G protein-coupled receptors, Islets of Langerhans, Insulin secretion, Alternative complement pathway, C3aR, C5aR1

## Abstract

**Aims:**

Complement components 3 and 5 (C3 and C5) play essential roles in the complement system, generating C3a and C5a peptides that are best known as chemotactic and inflammatory factors. In this study we characterised islet expression of C3 and C5 complement components, and the impact of C3aR and C5aR1 activation on islet function and viability.

**Materials and methods:**

Human and mouse islet mRNAs encoding key elements of the complement system were quantified by qPCR and distribution of C3 and C5 proteins was determined by immunohistochemistry. Activation of C3aR and C5aR1 was determined using DiscoverX beta-arrestin assays. Insulin secretion from human and mouse islets was measured by radioimmunoassay, and intracellular calcium ([Ca^2+^]i), ATP generation and apoptosis were assessed by standard techniques.

**Results:**

C3 and C5 proteins and C3aR and C5aR1 were expressed by human and mouse islets, and C3 and C5 were mainly localised to β- and α-cells. Conditioned media from islets exposed for 1 h to 5.5 and 20 mM glucose stimulated C3aR and C5aR1-driven beta-arrestin recruitment. Activation of C3aR and C5aR1 potentiated glucose-induced insulin secretion from human and mouse islets, increased [Ca^2+^]i and ATP generation, and protected islets against apoptosis induced by a pro-apoptotic cytokine cocktail or palmitate.

**Conclusions:**

Our observations demonstrate a functional link between activation of components of the innate immune system and improved β-cell function, suggesting that low-level chronic inflammation may improve glucose homeostasis through direct effects on β-cells.

**Electronic supplementary material:**

The online version of this article (doi:10.1007/s00018-017-2655-1) contains supplementary material, which is available to authorized users.

## Introduction

Type 2 diabetes (T2D) is a chronic disease characterised by increased insulin resistance and decreased β-cell mass and function [1]. The complement system, an important component of the innate immune system, consists of several small, inactive serum precursor proteins that are mainly synthesised by the liver. Upon activation, proteases cleave the precursor C3 and C5 into the active complement fragments C3a, C3b, C5a and C5b. These proteolytic cleavage cascades of intact C3 and C5 are responsible for the formation of a membrane attack complex and activation of an adaptive immune response [2]. The C3a and C5a peptide fragments that are generated, also known as anaphylatoxins, are responsible for production of local inflammatory responses and they exert at least part of their biological effects via activating the G protein-coupled receptors (GPCRs) C3aR, C5aR1 and C5aR2 that are encoded by the C3AR1, C5AR1 and C5AR2 genes [3–5].

In addition to their roles in innate immunity, an emerging body of literature suggests that intact C3 and C5 complement proteins, and the cleaved C3a and C5a peptides, are important in regulating whole body metabolism, energy homeostasis and the pathogenesis of diabetes and metabolic syndrome. For example, C3/C3a are positively associated with the incidence of diabetes [6–8] and insulin resistance [9, 10], and C5/C5a contribute to adipose tissue inflammation and insulin resistance [11, 12]. Moreover, C3a and C5a receptor antagonists have been reported to inhibit diet-induced obesity, metabolic dysfunction, and adipocyte and macrophage signalling in vivo in the rat [13]. In contrast, the adipokine adipsin (also known as complement factor D (CFD)), an adipose tissue-derived protease required for the generation of the C3 convertase, improves islet β-cell function by increasing conversion of circulating C3 into C3a [14]. Thus, studies to date implicate intact C3 and C5 and their peptide fragments in metabolic dysregulation through induction of insulin resistance, while a report with adipsin [14] suggests beneficial effects of C3a on β-cells. Since very little is currently known about the direct effects of C3/C3a and C5/C5a on β-cell function, the aims of this study were to determine the mRNA and protein expression of C3/C3a and C5/C5a in islets, and identify the effects of C3aR and C5aR1 activation on human and mouse islet secretory function and viability.

## Materials and methods

### Reagents

The C3aR agonist sc-214644, C3aR antagonist SB 290157 and C5aR1 agonist 65121-ANA were from Cambridge Bioscience (Cambridge, UK). The C5aR1 antagonists PMX 205 and W 54011 were from Tocris Bioscience (Bristol, UK). Intact C3 and C5 proteins were from Millipore (UK) Limited (Hertfordshire, UK), C3 antibody was from LifeSpan BioSciences, Inc. (Seattle, USA) and C5 antibody was from Insight Biotechnology (Wembley, UK). The glucagon antibody was from Sigma-Aldrich (Dorset, UK), insulin antibody from DAKO UK Ltd. (Ely, UK) and somatostatin antibody from Abcam plc (Cambridge, UK). Anti-rabbit, anti-guinea pig, anti-rat and anti-mouse secondary antibodies were from Jackson ImmunoResearch (Suffolk, UK). DAPI nucleic acid stain was from Thermo Fisher Scientific (Northumberland, UK). C3aR and C5aR1 beta-arrestin assays were from DiscoverX Corporation, Ltd. (Birmingham, UK). Recombinant murine TNFα, IFNγ and IL-1β were from PeproTech EC Ltd. (London, UK). Caspase 3/7 assay kits and CellTiter-Glo^®^ 3D assay kits were from Promega UK (Southampton, UK). The TaqMan RT-PCR kit was from Thermo Fisher Scientific (Loughborough, UK) and QuantiTect SYBR Green qPCR kits with QuantiTect qPCR assays were from Qiagen Ltd. (Manchester, UK). All other chemicals were from Sigma-Aldrich or Thermo Fisher Scientific.

### Human and mouse islet isolation and culture

Islets were isolated from 10–12-week-old male Crl:CD1 (ICR) mice (Charles River), by collagenase digestion of the exocrine pancreas [15]. Human islets were isolated from heart-beating non-diabetic donors, with appropriate ethical approval, at the King’s College London Human Islet Isolation Unit [16]. Islets were maintained overnight at 37 °C in culture medium supplemented with 5.6 mM glucose, 10% FCS, 2 mM glutamine and penicillin–streptomycin (100 U/mL, 0.1 mg/mL) before experimental use.

### RNA extraction and quantitative real-time PCR

A modified TRIzol protocol [17] was used to extract total RNA from human and mouse islets, and RNA was converted into cDNA using the TaqMan RT-PCR kit. Real-time PCR was performed using a LightCycler480 and gene expression relative to the house-keeping genes ACTB, GAPDH, PPIA, TBP and TCRF was carried out using QuantiTect qPCR assays (Supplementary Table 1), as described elsewhere [18, 19]. All complement and reference gene primer efficiency (E) [20] values were in the range of 1.85–2.15. For all complement and reference gene quantifications, template cDNAs were diluted in such a way that all quantified genes returned *C*_t_ values <30. Genes that expressed <0.001% of the mean level of the house keeping genes were considered to be present only at trace level, as their mRNA expression was below the lower limit of linear quantification for the QuantiTect primer assays.

### Immunohistochemistry

C3 and C5 complement protein expression by mouse and human pancreas was determined using rabbit anti-C3 and anti-C5 antibodies (both at 1:20) and Alexa Fluor^®^ 488 anti-rabbit secondary antibody (1:150). C3 and C5 protein localisation in islet cells was probed by co-staining with guinea pig anti-insulin (1:200), mouse anti-glucagon (1:100) and rat anti-somatostatin (1:50) and species-specific Alexa Fluor^®^ 594 secondary antibodies (all at 1:200). Nuclei were detected using DAPI (1:500). Specificity of immunofluorescent staining was confirmed by staining consecutive sections in the absence of primary antibody but with secondary antibody alone (at 1:150).

### Beta-arrestin assays

Induction of beta-arrestin recruitment for C3aR and C5aR1 was determined using PathHunter^®^ eXpress C3aR U2OS and C5aR1 CHO-K1 GPCR beta-arrestin kits. In brief, 8000 C3aR U2OS or C5aR1 CHO-K1 cells were dispensed into wells of the 96-well plate and incubated for 48 h (37 °C, 5% CO_2_). These cells were then exposed for 90 min to C3aR and C5aR1 agonists, intact C3 or C5 complement proteins or conditioned medium obtained from mouse islets that had been incubated for 1 h (37 °C, 5% CO_2_) in a physiological salt solution in the absence of glucose or in medium supplemented with 5.5 or 20 mM glucose. Beta-arrestin activity driven by C3aR and C5aR1 was quantified according to the manufacturer’s instructions using a luminescence plate reader on an unfiltered, full-range luminescence setting.

### Static insulin secretion

Groups of five mouse and human islets were incubated for 1 h in a physiological salt solution [21], either in the absence or presence of 1 µM C3aR or C5aR1 agonists or antagonists or with 100 nM intact C3 or C5 complement proteins (alone or in combination with 1 µM of the respective antagonist). In some experiments insulin secretion was quantified in the presence of increasing concentrations (1 nM–10 µM) of C3aR or C5aR1 antagonists SB 290157, PMX 205 or W 54011. Insulin secreted into the supernatant was quantified by radioimmunoassay, essentially as described elsewhere [22].

### Dynamic insulin secretion

The effects of agonists and antagonists at C3aR or C5aR1 on dynamic insulin secretion from human islets were assessed using a temperature-controlled perifusion system [23, 24]. Briefly, 50 human islets were transferred to chambers containing 1 μm filters and perifused (37 °C, 0.5 mL/min) with a salt solution [21] containing 2 or 20 mM glucose in the absence or presence of C3aR or C5aR1 agonists and/or antagonists. Perifusate samples were collected at 2-min intervals and secreted insulin was quantified by radioimmunoassay [22].

### ATP

Islet ATP production, which is a marker of islet metabolism, was quantified using the CellTiter-Glo^®^ 3D assay. 200 mouse islets were pre-incubated for 1 h in a salt solution [21] supplemented with 2 mM glucose then groups of 3 islets were incubated in 96-well plates in the absence or presence of intact C3 or C5 complement proteins or C3aR or C5aR1 agonists or antagonists. Cells were lysed and ATP was quantified according to the manufacturer’s instructions.

### Intracellular calcium ([Ca^2+^]i)

Groups of approximately 100,000 dispersed mouse islet cells were seeded onto glass coverslips, maintained in culture overnight then loaded with 5 μM Fura-2 AM for 30 min. Cells were perifused (37°C, 1 mL/min) with a physiological salt solution in the absence or presence of test agents and real-time changes in [Ca^2+^]i were determined by illuminating cells alternately at 340 and 380 nm, with emitted light being filtered at 510 nm.

### Apoptosis

Mouse and human islet apoptosis was assessed in the presence or absence of C3aR or C5aR1 agonists and/or antagonists using the Caspase-Glo 3/7 assay according to the manufacturer’s instructions. Anti-apoptotic effects were evaluated following induction of apoptosis by either palmitate (500 μM) or a cocktail of pro-apoptotic cytokines (25 U/mL IL-1β; 1000 U/mL TNFα; 1000 U/mL IFNγ).

### Statistical analysis

GraphPad Prism 5.0 (GraphPad Software, Inc) was used for statistical analysis. Data are presented as mean ± SEM and were analysed using ANalysis Of VAriance (ANOVA). Statistical significance was set at *p* values of <0.05 (*), <0.01 (**), <0.001 (***), <0.0001 (****).

## Results

### Quantification of complement component mRNA expression in human and mouse islets

**Fig. 1:**
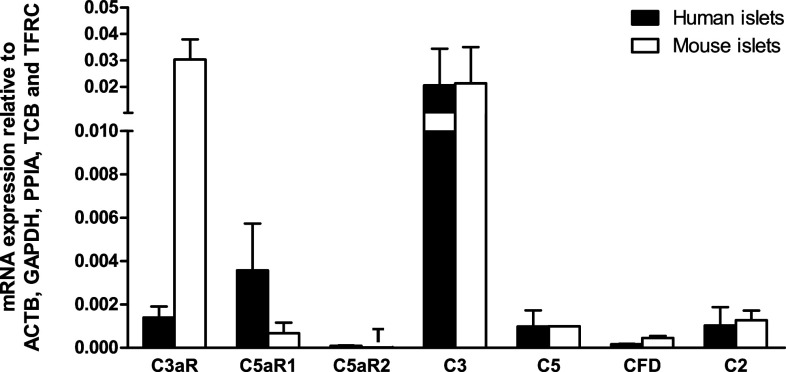
Relative mRNA expression of complement receptors and proteins by human and mouse islets. Data are presented as mean expression relative to the reference genes ACTB, GAPDH, PPIA, TBP and TFRC. *T* trace mRNA expression, *C3aR* complement receptor C3a, *C5aR1* complement receptor C5a1, *C5aR2* complement receptor C5a2, *C3* complement component 3, *C5* complement component 5, *CFD* complement factor D (adipsin), *C2* complement component 2, *n* = 3–4 individual human or mouse islet donors

Human and mouse islets expressed similar levels of mRNAs encoding intact C3 and C5 complement proteins, with C3 mRNA levels being approximately 20-fold higher than those of C5 (Fig. 1). The qPCR analysis also indicated that human and mouse islets expressed mRNAs encoding the receptors through which active C3a and C5a complement peptides exert their effects. Thus, C3aR mRNA was expressed at high levels in mouse islets and at low levels in human islets, whereas mRNA encoding C5aR1 was found at intermediate or low levels in human and mouse islets, respectively. C5a is also a ligand for C5aR2, but mRNA encoding this receptor was below the lower limit of linear quantification for the QuantiTect primer assays in both human and mouse islets. Complement factor D (CFD), also known as adipsin, and complement 2 (C2) help encoding complement cascade proteins with C3 and C5 convertase activity [25], which cleave C3 and C5 to generate the active C3a and C5a peptides. Both CFD and C2 mRNAs were detectable at low levels in human and mouse islets (Fig. 1).

### Immunohistochemical detection of C3 and C5 in mouse and human pancreas

**Fig. 2:**
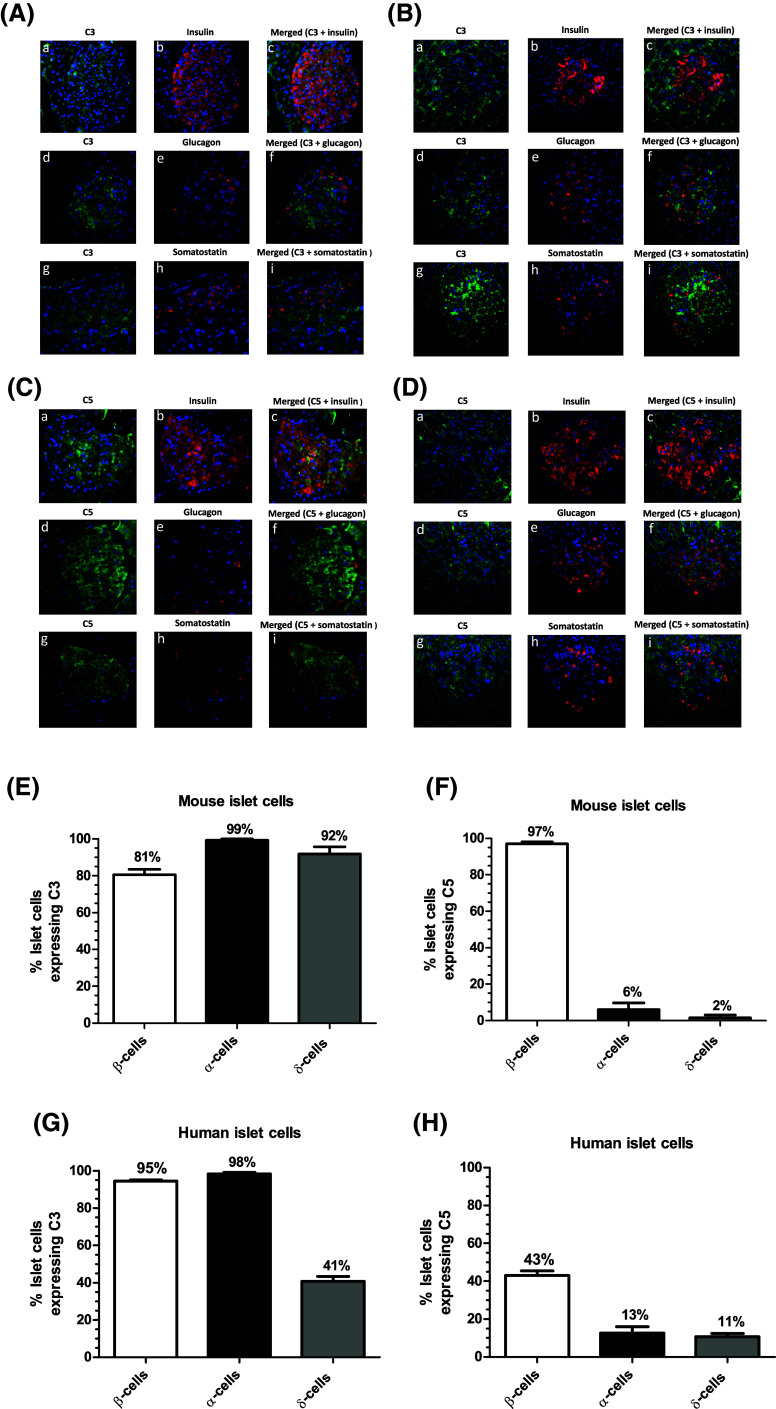
Expression of C3 and C5 complement proteins by mouse and human pancreas. **A**–**D** Co-localisation of C3 (**A**, **B**) and C5 (**C**, **D**) complement proteins (green) with the islet hormones insulin (*a*–*c*), glucagon (*d*–*f*) and somatostatin (*g*–*i*) (red) in mouse (**A**, **C**) and human (**B**, **D**) islets. Nuclei have been counterstained with DAPI (blue). C3 and C5 were also expressed by exocrine cells in both mouse and human pancreas. **E**, **G** C3 co-localises in mouse and human islets with insulin, glucagon and somatostatin, indicating expression of C3 in β-, α- and δ-cells. **F**, **H** C5 co-localises in mouse and human islets primarily with insulin, indicating predominant expression of C5 in β-cells

Fluorescence immunohistochemical staining indicated that C3 was distributed throughout mouse (Fig. 2A) and human (Fig. 2B) islets. Co-staining with antibodies against insulin, glucagon and somatostatin demonstrated that C3 co-localised with all three hormones, indicating that it is expressed by α-, β- and δ-cells (Fig. 2a, b, e, g). C5 was also detected in mouse (Fig. 2c) and human (Fig. 2d) islets, where it co-localised primarily with insulin, and low expression of this complement protein was also detected in α- and δ-cells (Fig. 2c, d, f, h). C3 and C5 proteins were also expressed by exocrine cells in both mouse and human pancreas (Fig. 2a–d).

### Quantification of C3aR and C5aR1 activation

**Fig. 3:**
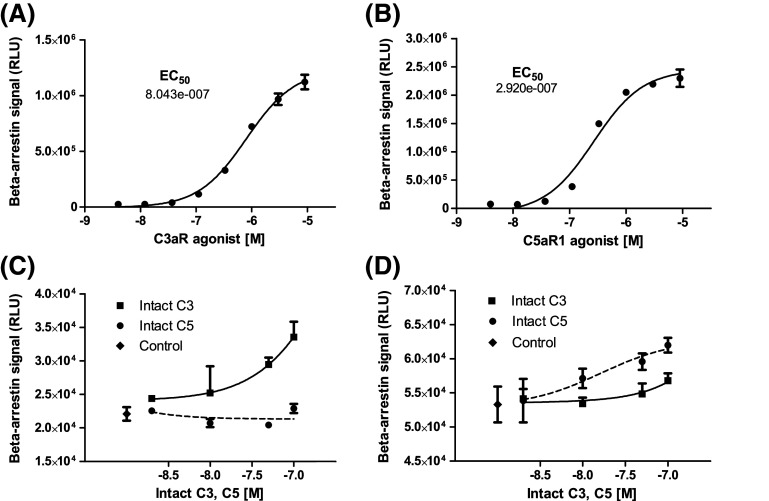
C3aR and C5aR1 beta-arrestin recruitment. **a**, **b** Concentration–response curves for high affinity C3aR (sc-214644) and C5aR1 (65121-ANA) agonists. **c** Intact C3 (solid line) induced concentration-dependent activation of C3aR, whereas intact C5 (dotted line) had no effect. **d** Intact C5 induced concentration-dependent activation of C5aR1 (dotted line), and very weak activation was also observed with intact C3 (solid line)

DiscoverX beta-arrestin reporter cell assays indicated that commercially available selective agonists for C3aR (sc-214644) and C5aR1 (65121-ANA) induced concentration-dependent activation of C3aR and C5aR1, with half-maximal activation at 804 nM sc-214644 and 292 nM 65121-ANA, respectively (Fig. 3a, b). The agonists were, therefore, used at 1 μM for islet functional experiments to ensure robust activation of C3aR and C5aR1. Intact C3 and C5 complement proteins also stimulated C3aR- and C5aR1-dependent beta-arrestin recruitment: C3 activated C3aR and, to a much lesser extent, C5aR1, whereas C5 only evoked beta-arrestin recruitment to C5aR1 (Fig. 3c, d).

### Islet-derived C3aR and C5aR1-activating factors

**Fig. 4:**
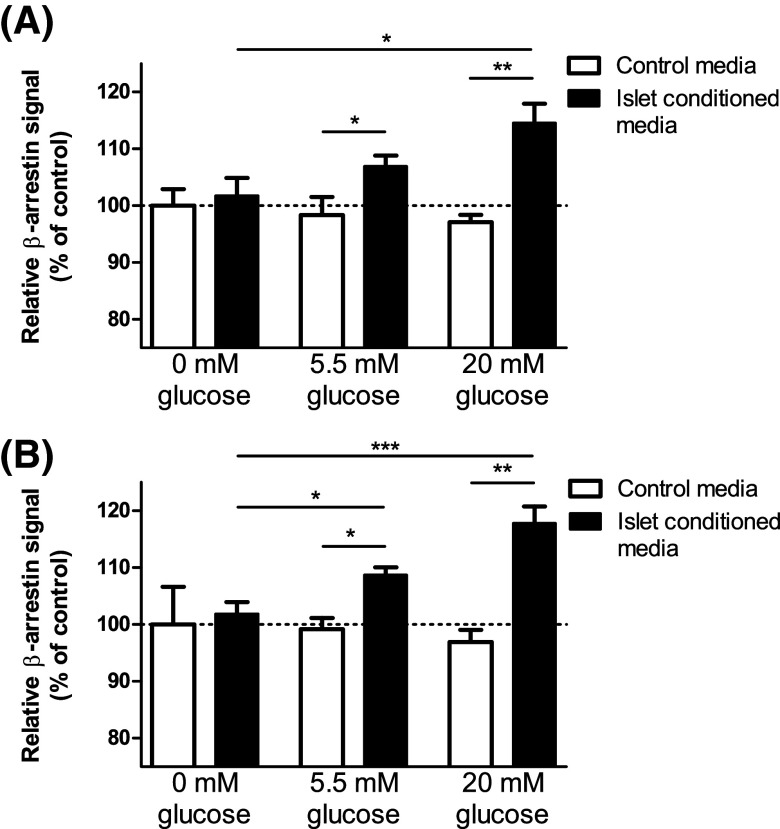
Islet-derived C3aR and C5aR1 activating factors. A glucose-dependent increase in beta-arrestin activity of C3aR (**a**) and C5aR1 (**b**) was observed in the presence of mouse islet-conditioned media, suggesting that C3aR and C5aR1-activating factors are released in a glucose-dependent manner from mouse islets. *p < 0.05, **p < 0.01, ***p < 0.001, *n* = 3–6

Since our qPCR and immunohistochemical data indicated that islets express intact C3 and C5, the beta-arrestin assays were also used to determine whether factors capable of activating C3aR and C5aR1 were released from islets. Conditioned media retrieved from mouse islets that had been incubated for 1 h stimulated C3aR (Fig. 4a) and C5aR1 (Fig. 4b) beta-arrestin recruitment in a glucose concentration-dependent manner, while medium from islets that had been incubated in the absence of glucose did not activate the receptors.

### Effects of modifying C3aR and C5aR1 activity on glucose-induced insulin secretion

**Fig. 5:**
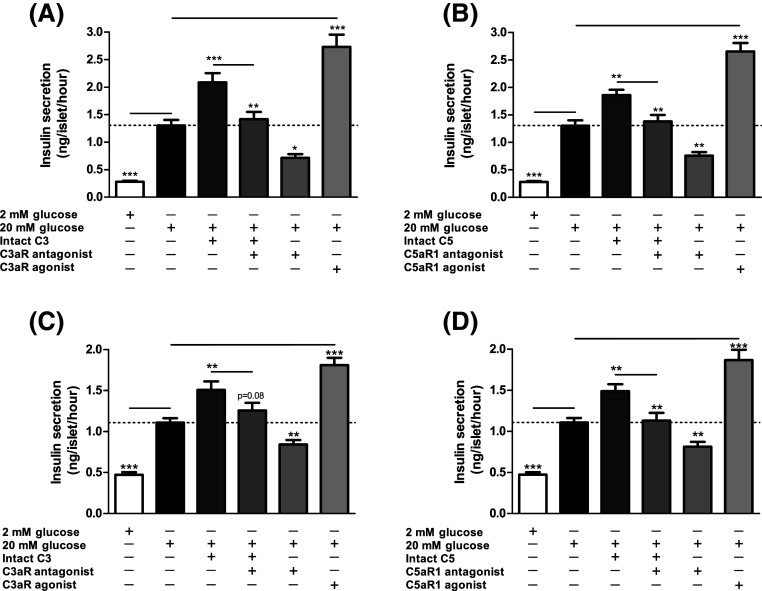
Effects of complement components on static insulin secretion in islets. Effects of C3aR agonist sc-214644, intact C3 and C3aR antagonist SB 290157 (**a**, **c**) and C5aR1 agonist 65121-ANA, intact C5 and C5aR1 antagonist PMX 205 (**b**, **d**) on glucose-stimulated insulin secretion from mouse (**a**, **b**) and human (**c**, **d**) islets. C3aR (1 µM, sc-214644) and C5aR1 (1 µM, 65121-ANA) agonists potentiated glucose-induced insulin secretion from mouse and human islets, with similar stimulatory effects observed in islets incubated with 100 nM intact C3 and C5. Stimulation by intact C3 and C5 was blocked by SB 290157 and PMX 205, which also inhibited glucose-induced insulin secretion. *p < 0.05, **p < 0.01, ***p < 0.001, *n* = 15

**Fig. 6:**
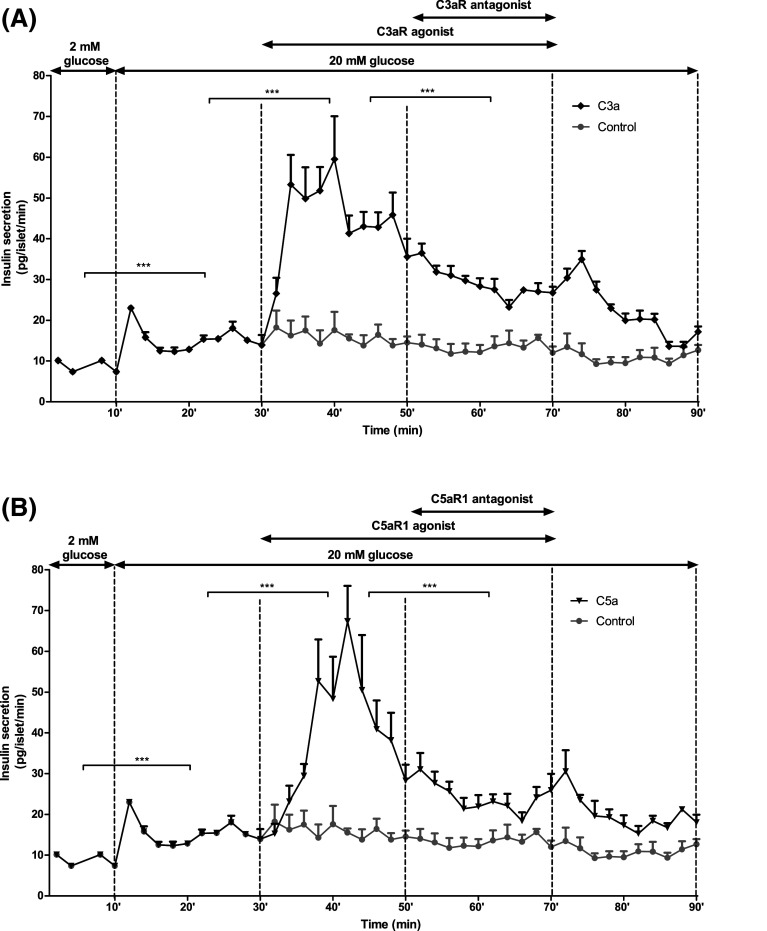
Effects of complement components on time-resolved insulin secretion from human islets. Addition of sc-214644 (**a**) or 65121-ANA (**b**) to a physiological buffer significantly increased glucose-stimulated insulin secretion from human islets, whereas addition of SB 290157 or PMX 205 in the presence of the corresponding agonist significantly decreased insulin secretion. ***p < 0.001, *n* = 4

Exposure of mouse and human islets to C3aR (1 μM, sc-214644) and C5aR1 (1 μM, 65121-ANA) agonists resulted in significant potentiation of glucose-induced insulin secretion, and stimulatory effects were also observed when islets were incubated with intact C3 and C5 (Fig. 5a–d). Potentiation of insulin secretion by intact C3 and C5 was reduced by co-incubation of islets with C3aR and C5aR1 antagonists, which also inhibited insulin secretion stimulated by 20 mM glucose alone (Fig. 5a–d). This inhibition of glucose-stimulated insulin secretion by C3aR and C5aR1 antagonists was confirmed by constructing concentration–response curves using mouse islets, which indicated half-maximal inhibition at ~45–80 nM of antagonists and maximal inhibition at 1 μM (Supplementary Figure 1A–C). Based on these results, antagonists were used in functional experiments at 1 µM to maximally inhibit C3aR and C5aR1. Time-resolved perifusion experiments with human islets confirmed the capacity of sc-214644 (Fig. 6a) and 65121-ANA (Fig. 6b) to potentiate glucose-induced insulin secretion. The elevation in insulin release with both agonists was rapid in onset and insulin secretion was significantly reduced in the presence of SB 290157 and PMX 205.

### Effects of modifying C3aR and C5aR1 activity on islet ATP generation

**Fig. 7:**
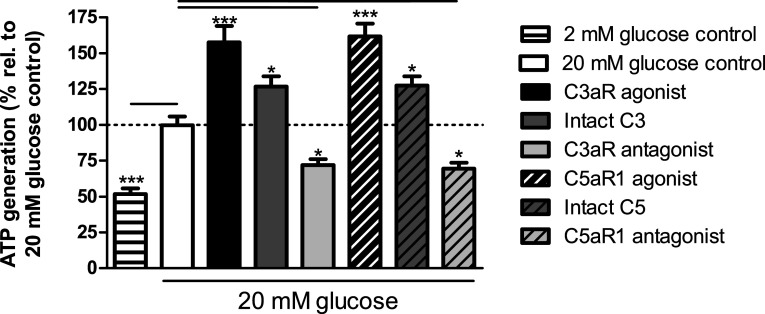
ATP generation stimulated by complement components in mouse islets. sc-214644, 65121-ANA and intact C3 and C5 significantly stimulated ATP production in mouse islets, whereas SB 290157 and PMX 205 significantly reduced glucose-induced ATP generation. *p < 0.05, ***p < 0.001, *n* = 12

Quantification of islet ATP generation indicated that C3aR activation with 1 μM sc-214644 significantly elevated ATP generation at 20 mM glucose, and similar effects were obtained following C5aR1 activation by 1 μM 65121-ANA (Fig. 7). Exposure of mouse islets to 100 nM intact C3 or C5 also significantly potentiated glucose-stimulated ATP production while C3aR and C5aR1 antagonists significantly inhibited ATP generation at 20 mM glucose (Fig. 7).

### Effects of C3aR and C5aR1 agonists on islet cell intracellular Ca^2+^ levels

**Fig. 8:**
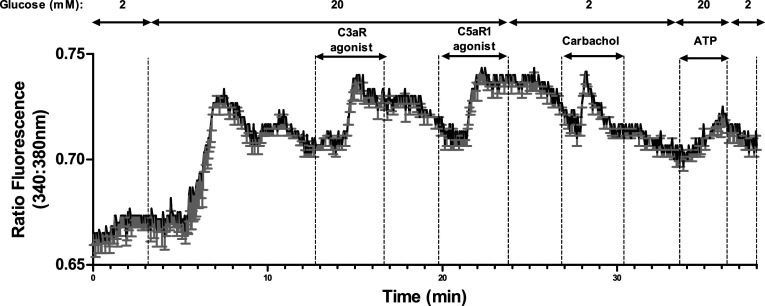
Stimulatory effects of C3aR and C5aR1 agonists on intracellular calcium in mouse islets. C3aR (1 µM, sc-214644) and C5aR1 (1 µM, 65121-ANA) agonists reversibly elevated [Ca^2+^]i in mouse islet cells. 500 µM carbachol was used as a positive control. Mean changes in [Ca^2+^]i levels in six individual cells, expressed as the 340 nm/380 nm fluorescence ratio, are shown in black and the SEMs for each time point are shown in grey

C3a and C5a are reported to elevate [Ca^2+^]i in neutrophils [26, 27] so Fura-2-loaded mouse islets were used to identify whether islet C3aR and C5aR1 were coupled to increases in [Ca^2+^]i. Agonists at C3aR (1 μM sc-214644) and C5aR1 (1 μM 65121-ANA) caused rapid, sustained elevations in [Ca^2+^]i at 20 mM glucose, which were reversible following removal of the activating ligand, and the islet cells also responded to 500μM carbachol with the expected increase in [Ca^2+^]i (Fig. 8).

### Effects of C3aR and C5aR1 activation on islet apoptosis

**Fig. 9:**
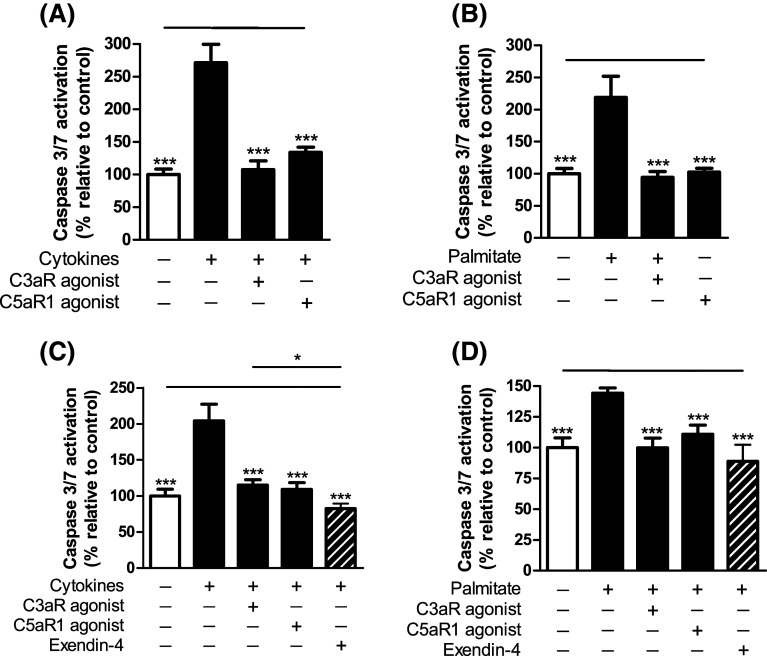
Effects of C3aR and C5aR1 agonists on mouse and human islet apoptosis. Caspase 3/7 activities in mouse (**a**, **b**) and human (**c**, **d**) islets were induced by a cocktail of pro-apoptotic cytokines (**a**, **c**) or by palmitate (**b**, **d**). sc-214644 and 65121-ANA protected against apoptosis in both mouse and human islets. 20 nM Exendin-4 was included as reference treatment for the human islet experiments (**c**, **d**). ***p < 0.001, *n* = 6

Mouse and human islet caspase 3/7 activation, induced either by a cocktail of pro-apoptotic cytokines or by the saturated fatty acid palmitate, was significantly inhibited in the presence of C3aR and C5aR1 agonists (Fig. 9a–d). In human islets, the protection against palmitate-induced apoptosis by C3aR and C5aR1 activation was of a similar magnitude to that of 20 nM Exendin-4, an established anti-apoptotic GPCR agonist in islets.

## Discussion

The complement system consists of a tightly regulated network of proteins that play important roles in host defence and inflammation. Intact C3 and C5 proteins and the cleaved C3a and C5a peptides are reported to act as mediators of inflammation-induced insulin resistance [9–12], but a beneficial effect of complement factor D, a protease required for the generation of the C3 convertase, on β-cell function has been demonstrated [14]. Here, we have demonstrated C3 and C5 mRNA and protein expression by human and mouse islets, and that C3aR and C5aR1 activation promotes improvements in islet secretory function and viability.

Our qPCR analysis showed that genes for all complement components studied were present in mouse and human islet preparations. The identification of C3 mRNA in ICR mouse islets, at levels at least tenfold higher than those of C5, is in contrast to an earlier report that C3 mRNA is not expressed by islets isolated from C57 BL/6 mice [14]. The reasons for this discrepancy are not immediately obvious, but may reflect strain-dependent differences in complement protein expression by the pancreas, as we have recently reported for GPCR expression [28]. Our quantification of C3 mRNA in islets from human donors, at levels similar to those identified in ICR mouse islets, also points to its expression by islet cells. However, since mRNA quantifications do not always reflect relative protein expression levels nor importance in a particular tissue [29], we used fluorescence immunohistochemistry to confirm expression of C3 and C5 in mouse and human islets, consistent with a functional role for these complement proteins in islet physiology. We also observed expression of complement proteins by exocrine cells, supporting an earlier report of local production of C3 in the exocrine pancreas where it was implicated in complement-mediated immunological protection [30]. We attempted to identify C3aR and C5aR1 expression by mouse and human islets, but we were unable to demonstrate specific staining with commercially available antibodies, consistent with low detection levels obtained by qPCR. Nonetheless, the functional effects of the C3aR and C5aR1 activation described in this paper indirectly indicate the presence of these receptors on islet cells.

Traditional assays for GPCRs identify the functional responses elicited by receptor activation and downstream signalling through heterotrimeric G proteins. Beta-arrestin recruitment, which occurs independently of G-protein coupling, is a powerful screening platform for direct measurement of GPCR activity by detecting interaction of beta-arrestin with the activated GPCR [31]. We took advantage of C3aR and C5aR1 beta-arrestin assays to demonstrate that islets secreted factors that stimulated C3aR and C5aR1-mediated beta-arrestin recruitment in a glucose-dependent manner. To the best of our knowledge, this is the first time that a cell-based beta-arrestin assay has been used to detect GPCR-activating factors released from islets. The specificity of these assays relies on the use of cell lines in which a small enzyme fragment is fused to the GPCR under investigation and a larger enzyme fragment is fused to beta-arrestin. When the receptor is activated, it binds to beta-arrestin and both enzyme fragments combine, resulting in enzyme activity that is detected by chemiluminescence. The beta-arrestin assays were also used to demonstrate that C3aR and C5aR1 agonists caused concentration-dependent activation of the receptors, as expected. Intact C3 and C5 were included in the beta-arrestin assays as negative controls since they are not thought to be agonists of the complement receptors. However, we unexpectedly found that exogenous intact C3 and C5 proteins stimulated low-level beta-arrestin recruitment, indicative of C3aR and C5aR1 activation, respectively. These data suggest that there was local production of the active C3a and C5a peptides in these experiments, either through expression of the corresponding convertases by the reporter U2OS and CHO-K1 cells or by proteases in the serum-supplemented medium, in accordance with published studies of spontaneous C3 activation in plasma [32, 33]. It cannot, however, be ruled out that intact C3 and C5 can act as low-affinity, direct activators of C3aR and C5aR1.

Plasma levels of C3 are positively associated with insulin secretion in non-diabetic human subjects, which may suggest that C3 is a contributor to insulin release [34]. Stimulatory effects on insulin secretion from mouse islets have been reported for acylation stimulating protein, a desArg derivative of C3a [35] and C3a itself, effects mediated by activation of islet C3aR [14]. Building on these earlier studies we have now observed that C3aR and C5aR1 agonists potentiated glucose-stimulated insulin secretion from both human and mouse islets, consistent with C3aR and C5aR1 being key positive regulators of insulin secretion. Similar stimulatory effects were observed with intact C3 and C5, suggesting that they are cleaved to the active peptides by their convertases, or that the proteins may themselves act as low-affinity direct activators of their corresponding receptors. Intact C3 and C5 were used for these experiments at 100 nM, which is lower than circulating concentrations of these complement components [36]. However, we have shown here that there is local production of these proteins by islets and it is possible that autocrine or paracrine signalling within islets occurs at lower concentrations than are reached during inflammation-mediated elevations in circulating intact C3 and C5. Antagonists of C3aR (SB 290157) and C5aR1 (PMX 205 and W 54011) induced concentration-dependent inhibition of glucose-stimulated insulin secretion, even in the absence of exogenously added C3aR and C5aR1 agonists. This in vitro inhibition of insulin secretion suggests that the endogenous islet-derived complement C3/a and C5/a peptides normally act in an auto/paracrine manner when released from islets to promote insulin release. These results are also in agreement with an earlier study in which antagonism of C3aR in *db/db* mice with SB 290157 inhibited adipsin-mediated improvements in glucose tolerance and insulin secretion [14].

It is well established that glucose metabolism and ATP synthesis in β-cells is essential for Ca^2+^ influx and insulin granule exocytosis [37]. C3aR and C5aR1 are pertussis toxin-sensitive GPCRs [38, 39], suggesting signalling through Gi that does not fit with their stimulatory effects on insulin release. However, it has been reported that C3aR activation can elevate [Ca^2+^]i [14], perhaps via Gq coupling. We found that C3aR and C5aR1 agonists significantly increased [Ca^2+^]i in mouse islets and they also led to significant elevations in ATP generation, as did intact C3 or C5. These observations suggest that islet C3aR- and C5aR1-mediated elevations in [Ca^2+^]i may lead to increased mitochondrial [Ca^2+^] [40], promoting Krebs cycle activity and leading to enhanced ATP content. The C3aR and C5aR1 antagonists significantly inhibited glucose-stimulated ATP production, which may have been responsible, at least in part, for their inhibitory effects of glucose-stimulated insulin secretion.

A major feature of T2D is the progressive loss of β-cell mass, reflecting a shift from islet quiescence/proliferation to β-cell apoptosis [1]. An anti-apoptotic effect of a C5aR1 agonist in neurons has been reported [41], an unexpected function for an anaphylatoxin. Further evidence of a protective role for complement peptides is provided by a recent study in which C3a and C5a peptides reduced apoptosis of myeloid and lymphoid cells [42]. In the current study, activation of mouse and human islet C3aR and C5aR1 led to a robust reduction in apoptosis that had been induced by a cytokine cocktail or the saturated fatty acid palmitate. This apparent paradoxical effect of complement cascade components may be a protective mechanism to maintain β-cell mass in conditions such as obesity, which normally exerts increased stress on the β-cell population. These findings also support previous proposals of a beneficial role of complement proteins on tissue remodelling [43].

In summary, our data reveal that C3 and C5 complement proteins and their receptors are expressed by human and mouse islets, and that islets secrete complement receptor activating ligands in a glucose-dependent manner. Activation of islet C3aR and C5aR1 results in increased intracellular calcium and ATP levels, potentiation of glucose-induced insulin secretion and protection against apoptosis. These observations demonstrate a functional link between activation of components of the innate immune and improved human and mouse β-cell function, suggesting that complement peptides exert direct stimulatory effects at islet β-cells that could compensate for their induction of peripheral insulin resistance.

### Electronic supplementary material

Below is the link to the electronic supplementary material.

**Supplementary Figure 1**. Effects of C3aR (A) and C5aR1 (B, C) receptor antagonists on glucose-stimulated insulin secretion. Exposure of mouse islets at 20 mM glucose to 1 nM-10 µM of C3aR antagonist SB 290157 (A) or C5aR1 antagonists PMX 205 (B) or W 54011 (C) inhibited glucose-stimulated insulin secretion in a concentration-dependent manner. Exposure of mouse islets to 1 µM SB 290157 (A), 1 µM PMX 205 (B) or 1μM W 54011 (C) also inhibited glucose-stimulated insulin secretion. Insulin secretion data are normalised to the insulin secretory response at 20 mM glucose. *: p < 0.05; **: p < 0.01; ***: p < 0.001; ****: p < 0.0001. n = 8 (PDF 686 kb)

**Supplementary Table 1**. qPCR primers used to quantify gene expression relative to ACTB, GAPDH, PPIA, TBP and TFRC in human and mouse islets (PDF 312 kb)
