# Supplementary figures and images for: C3aR and C5aR1 act as key regulators of human and mouse β-cell function
Source: Cell Mol Life Sci. 2017 Sep 18;75(4):715–26. doi: 10.1007/s00018-017-2655-1 (PMC5769825; doi:10.1007/s00018-017-2655-1)

## Supplementary Figure 1

A)

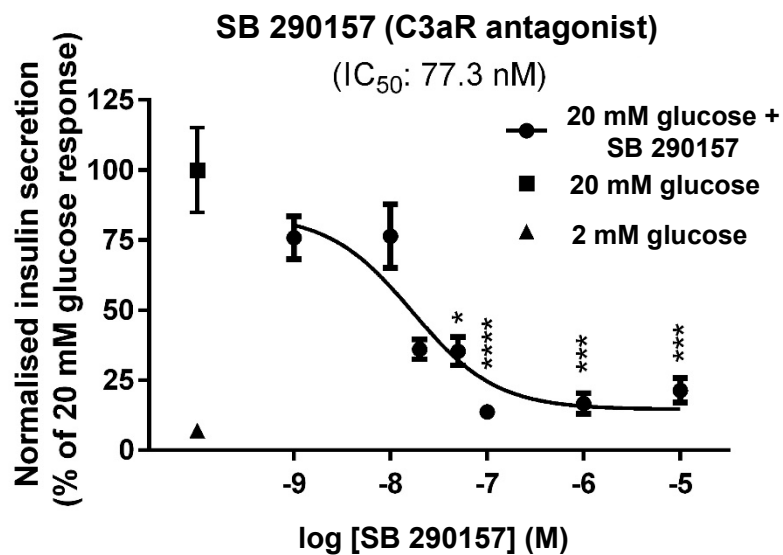

B)

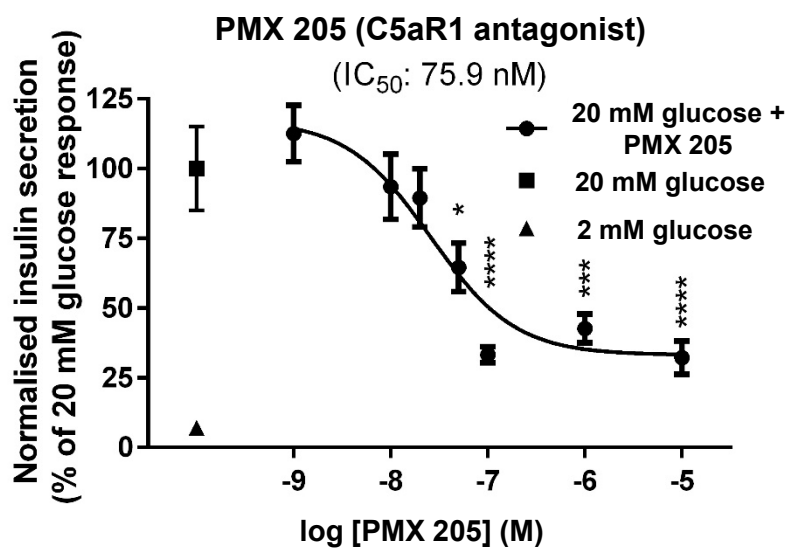

C)

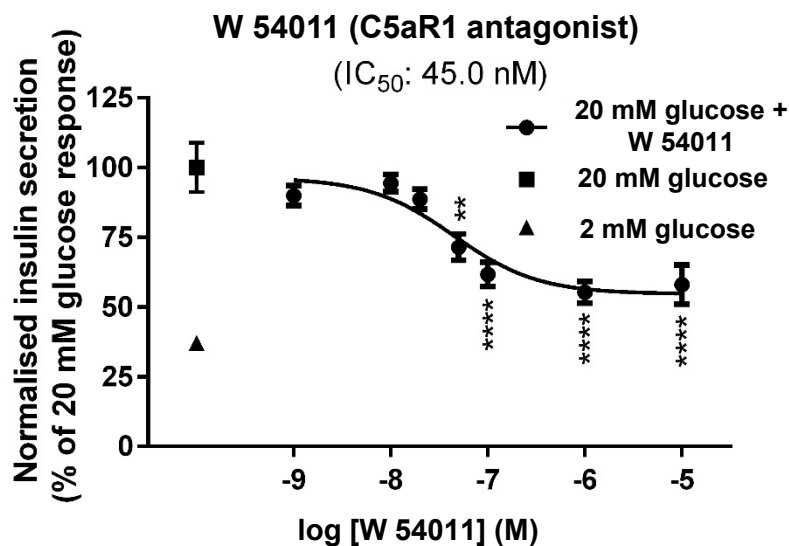

Supplement: Supplementary file 1 — Supplementary Figure 1. Effects of C3aR (A) and C5aR1 (B, C) receptor antagonists on glucose-stimulated insulin secretion. Exposure of mouse islets at 20 mM glucose to 1 nM-10 µM of C3aR antagonist SB 290157 (A) or C5aR1 antagonists PMX 205 (B) or W 54011 (C) inhibited glucose-stimulated insulin secretion in a concentration-dependent manner. Exposure of mouse islets to 1 µM SB 290157 (A), 1 µM PMX 205 (B) or 1μM W 54011 (C) also inhibited glucose-stimulated insulin secretion. Insulin secretion data are normalised to the insulin secretory response at 20 mM glucose. *: p < 0.05; **: p < 0.01; ***: p < 0.001; ****: p < 0.0001. n = 8 (PDF 686 kb) [file 18_2017_2655_MOESM1_ESM.pdf]
