# Supplementary material for: C3aR and C5aR1 act as key regulators of human and mouse β-cell function
Source: Cell Mol Life Sci. 2017 Sep 18;75(4):715–26. doi: 10.1007/s00018-017-2655-1 (PMC5769825; doi:10.1007/s00018-017-2655-1)
Supplement: Supplementary file 2 — Supplementary Table 1. qPCR primers used to quantify gene expression relative to ACTB, GAPDH, PPIA, TBP and TFRC in human and mouse islets (PDF 312 kb) [file 18_2017_2655_MOESM2_ESM.pdf]

Supplementary Table 1

Gene symbol	Human primer assay	Mouse primer assay
C3aR1	QT01676941	QT02380581
C5aR1	QT00997766	QT01164723
C5aR2	QT00243971	QT02532803
C3	QT01680413	QT00109270
C5/Hc	QT00088011	QT00102032
CFD	QT00212191	QT01051890
C2	QT00059024	QT00159152
ACTB	QT01680476	QT01136772
GAPDH	QT01192646	QT01658692
PPIA	QT01866137	QT00247709
TBP	QT00000721	QT00198443
TFRC	QT00094850	QT00122745
